# Expression of genes involved in DNA repair and cell cycle checkpoint pathways in Triple Negative compared to Luminal A Breast Cancer: a molecular characterization

**DOI:** 10.1186/1897-4287-10-S2-A37

**Published:** 2012-04-12

**Authors:** Monica Ganzinelli, Enilze Ribeiro, Ramona Bertoni, Letizia Bazzola, Daniele Andreis, Alberto Bottini, Roberto Giardini, Camillo Rossi, Giovanna Damia, Daniele Generali

**Affiliations:** 1 Istituto di Ricerche Farmacologiche Mario Negri Milan Italy, Ospedale San Gerardo, Universita’ di Milano Bicocca Monza, Italy; 2 Departamento de Genética, Setor de Ciencias Biologicas, Universidade Federal do Paraná, Rua Francisco H. dos Santos, Curitiba, Paranà, Brasil; 3 U.O. di Anatomia Patologica, Istituti Ospitalieri di Cremona, Cremona, Italy; 4 U.O. Multidisciplinare di Patologia Mammaria, Laboratorio di Oncologia Molecolare Senologica, Istituti Ospitalieri di Cremona, Cremona, Italy; 5 Direzione Sanitaria, Istituti Ospitalieri di Cremona, Cremona, Italy

## Purpose

Considering the clear emerging role of the DNA repair and the cell cycle checkpoints as predictive, prognostic and therapeutic targets in cancer there is a need to better characterized human tumours to define sub-sets of patients that would benefit of a particular treatment modality. The aim of the present work is to characterize molecularly a cohort of Triple Negative Breast Cancers (TNBC; estrogen receptor negative, progesterone receptor negative, and HER2/neu negative by immunohistochemistry) compared to Luminal A Breast Cancer (LABC; estrogen receptor positive and/or progesterone receptor positive, and HER2/neu negative, ki67 expression < 15% by immunohistochemistry) as regard of gene expression involved in DNA repair pathway (in particular genes involved in nucleotide excision repair, base excision repair, homologous recombination repair and BRCA/Fanconi anemia pathway) and cell cycle checkpoint pathway (CHK1) and their correlation with the clinical-pathological characteristics.

## Experimental design

A single core from archived clinically annotated tumor specimens from 80 women with TNBC and 70 with LABC were performed using Tissue Micro Array machine. The mRNA expression by RT-PCR of in genes involved in NER pathway (ERCC1, XPA, XPG), in the FA/BRCA pathway (FANC-A, FANC-C, FANC-F, FANC-D2), in BER pathway (PARP) and cell cycle checkpoint (Chk1) were analyzed by RT-PCR. Scores of single genes were combined with clinical data to assess association with outcome (Overall Survival –OS- and Event Free Survival –PFS).

## Results

Among the NER genes, ERCC1 (p < 0.0001) and XPA (p = 0.03) genes were significantly less expressed in TNBC than in LABC; among the FA genes, BRCA1 (p < 0.0001), FANCD2 (p < 0.0001), FANCF (p < 0.0001) and PALB2 (p = 0.0006) genes were significantly less expressed in TNBC than in LABC. Also CHK1 was less expressed in TNBC (p < 0.0001).

In relation to the clinical-pathological characteristics, lower level of XPG and FANCA were associated with larger tumor size (≥ pT2) at definitive surgery in TNBC.

The high expression of FANCA was related to an increase of either the Overall Survival (p = 0.0045) or the Event-Free Survival (p = 0.0141) on univariate analysis in TNBC.

## Conclusions

DNA repair and cell cycle checkpoint related genes with particular regards to ERCC1/XPA in NER family, BRCA/FANC family and CHK1 may be useful as prognostic markers in TNBC and likely to be important in familial BRCA mutated cancers accordingly to their affinity. Their determination could be relevant for clinicians in selecting the proper treatment to adopt in TNBC.

